# Supplementary material for: Application of TGA/c-DTA for Distinguishing between Two Forms of Naproxen in Pharmaceutical Preparations
Source: Pharmaceutics. 2023 Jun 8;15(6):1689. doi: 10.3390/pharmaceutics15061689 (PMC10303704; doi:10.3390/pharmaceutics15061689)
Supplement: Supplementary file 1 [file pharmaceutics-15-01689-s001.zip › pharmaceutics-2381527-supplementary.pdf]

SUPPLEMENTARY MATERIALS

Application of TGA/c-DTA for Distinguishing Between Two Forms of Naproxen in Pharmaceutical Preparations

Paweł Ramos [1]*, Barbara Klaudia Raczak[2,3], Daniele Silvestri[2], Stanisław Wacławek[2]

[1] Department of Biophysics, Faculty of Pharmaceutical Sciences in Sosnowiec, Medical University of Silesia in Katowice, Jedności 8, 41-200 Sosnowiec, Poland; pawelramos@sum.edu.pl (P.R.)

[2] Institute for Nanomaterials, Advanced Technologies and Innovation, Technical University of Liberec, Stdentská 2, 460 01 Liberec, Czech Republic; stanislaw.waclawek@tul.cz (S.W.), barbara.klaudia.raczak@tul.cz (B.K.R.), daniele.silvestri@tul.cz (D.S.)

[3] Faculty of Mechatronics, Informatics and Interdisciplinary Studies, Technical University of Liberec, 461 17 Liberec, Czech Republic; barbara.klaudia.raczak@tul.cz (B.K.R.)

*Correspondence: pawelramos@sum.edu.pl; Tel.: +480323641164

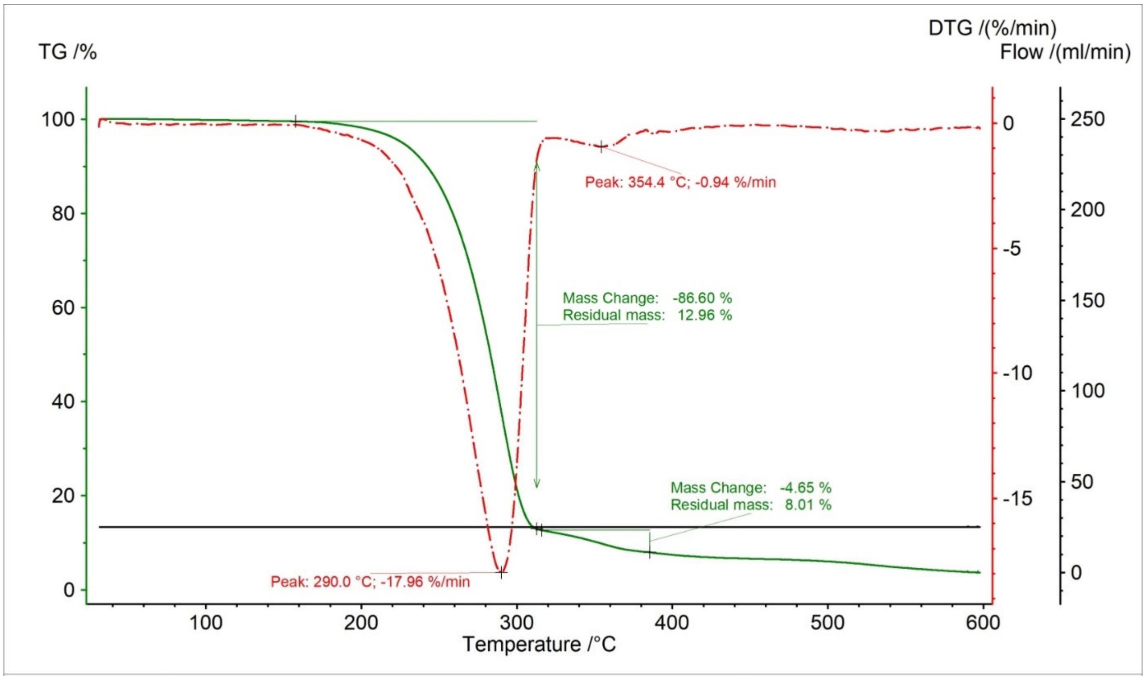

Figure S1. TG (green) and DTG (reed) curves of naproxen acid standard (NAS).

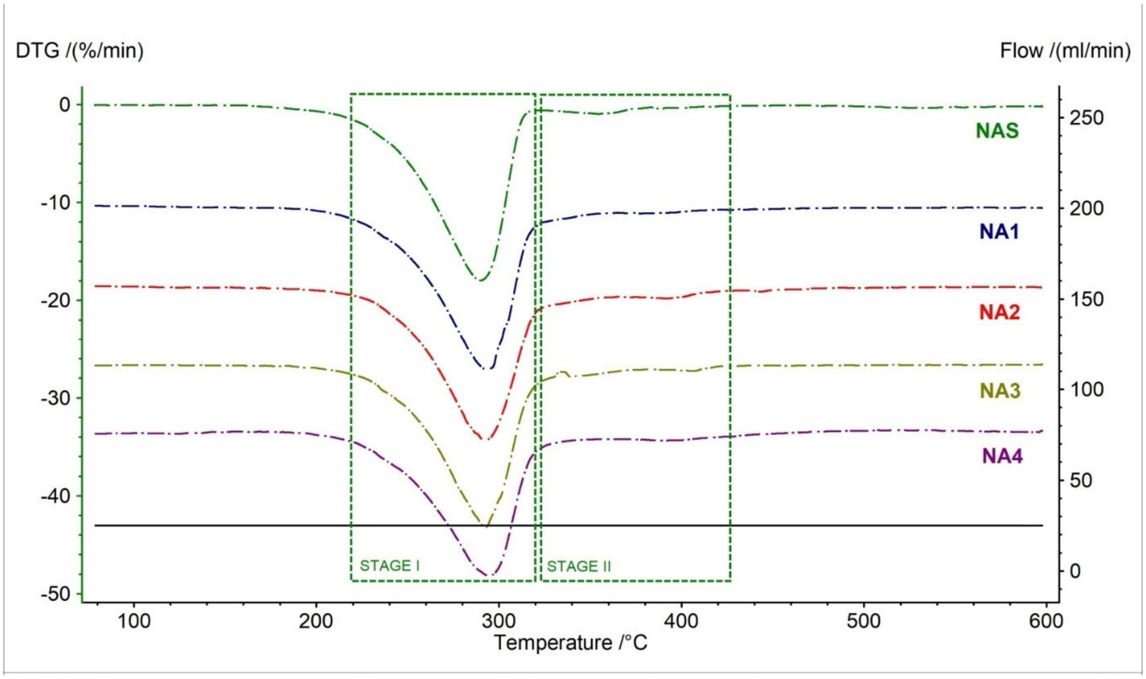

Figure S2. DTG curves of naproxen acid standard (NAS), and pharmaceutical preparations containing naproxen acid (NA1-NA4). The green frame shows the stages of decomposition related to the API.

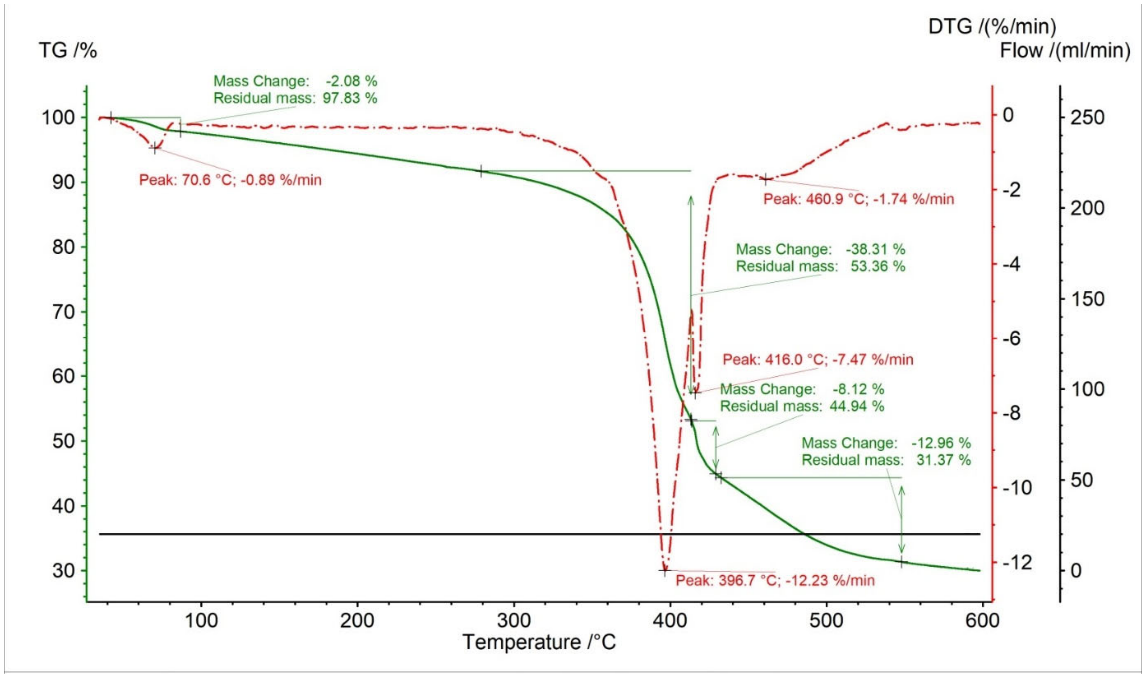

Figure S3. TG (green) and DTG (reed) curves of naproxen sodium standard (NSS).

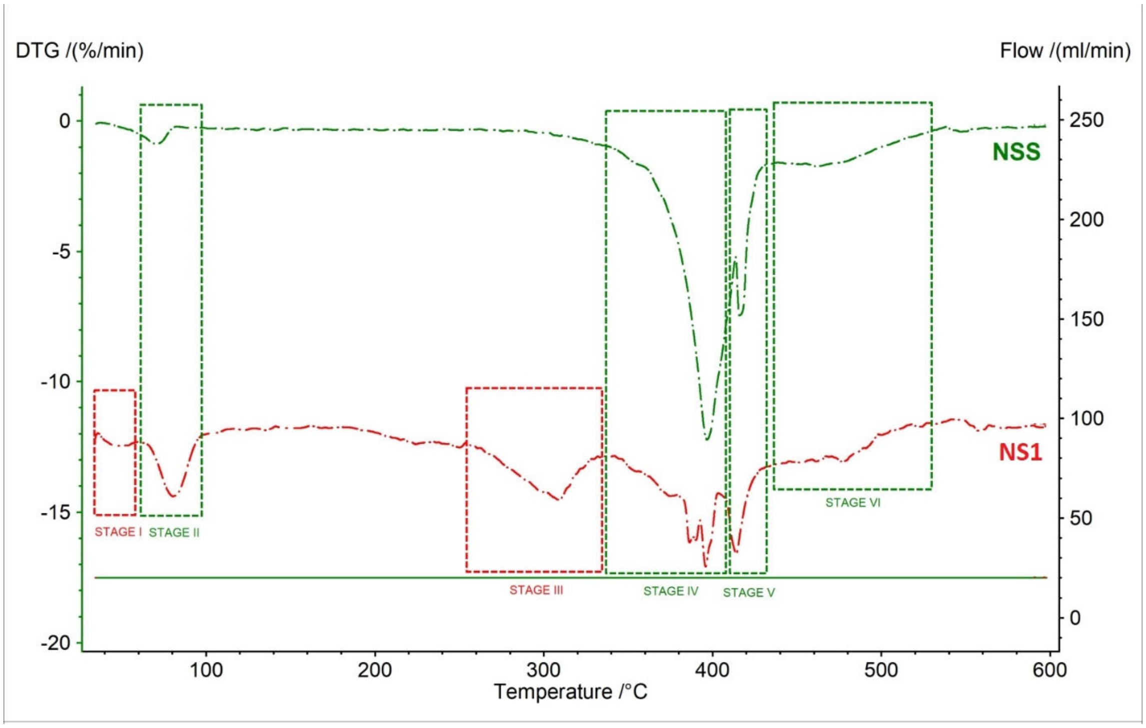

Figure S4. DTG curves of naproxen sodium standard (NSS), and pharmaceutical preparation containing naproxen sodium (NS1). The green frame shows the stages of decomposition related to the API, and the red frame - is associated with the excipient.

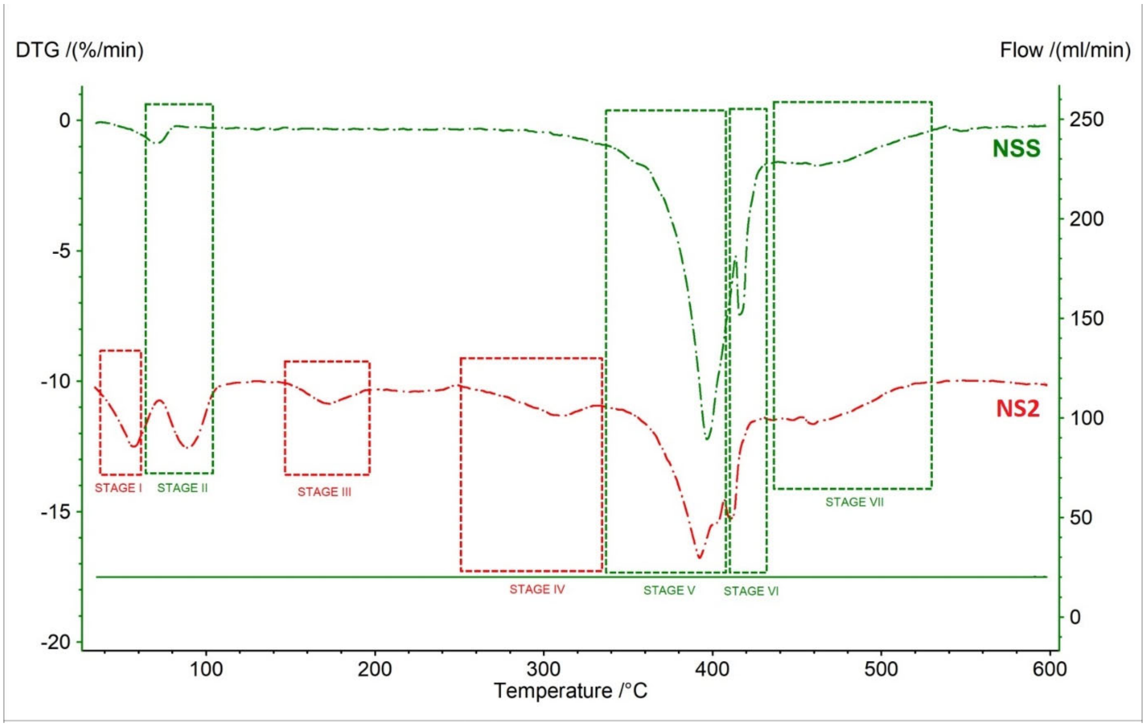

Figure S5. DTG curves of naproxen sodium standard (NSS), and pharmaceutical preparation containing naproxen sodium (NS2). The green frame shows the stages of decomposition related to the API, and the red frame - is associated with the excipient.

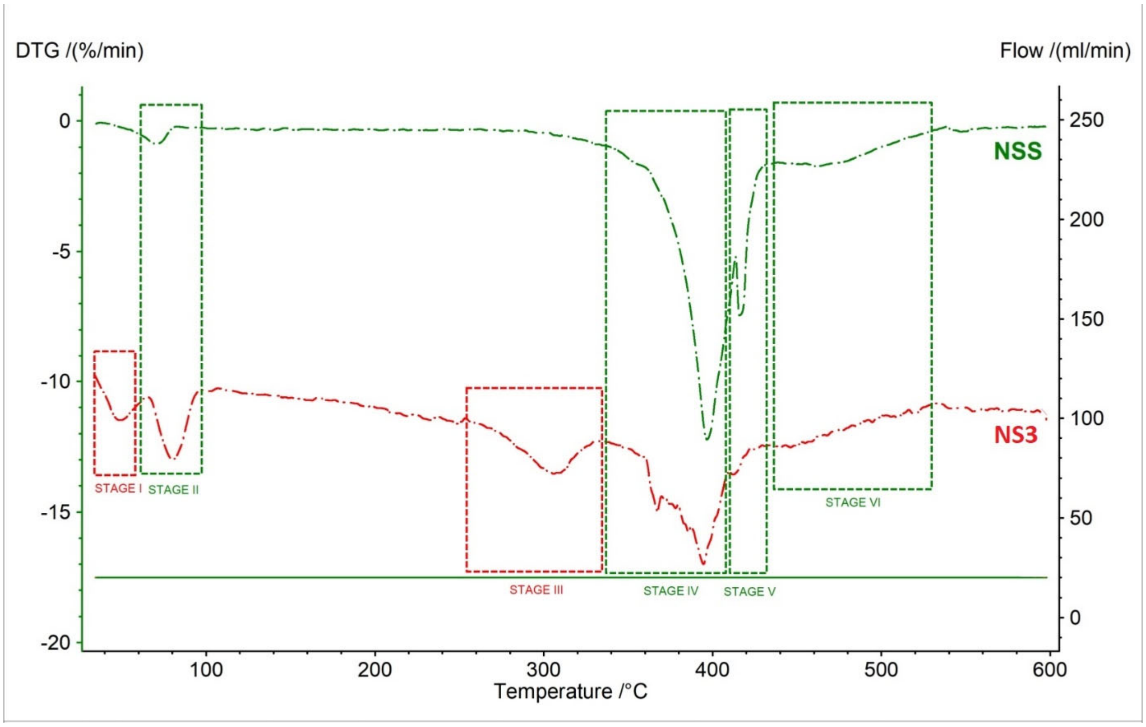

Figure S6. DTG curves of naproxen sodium standard (NSS), and pharmaceutical preparation containing naproxen sodium (NS3). The green frame shows the stages of decomposition related to the API, and the red frame - is associated with the excipient.

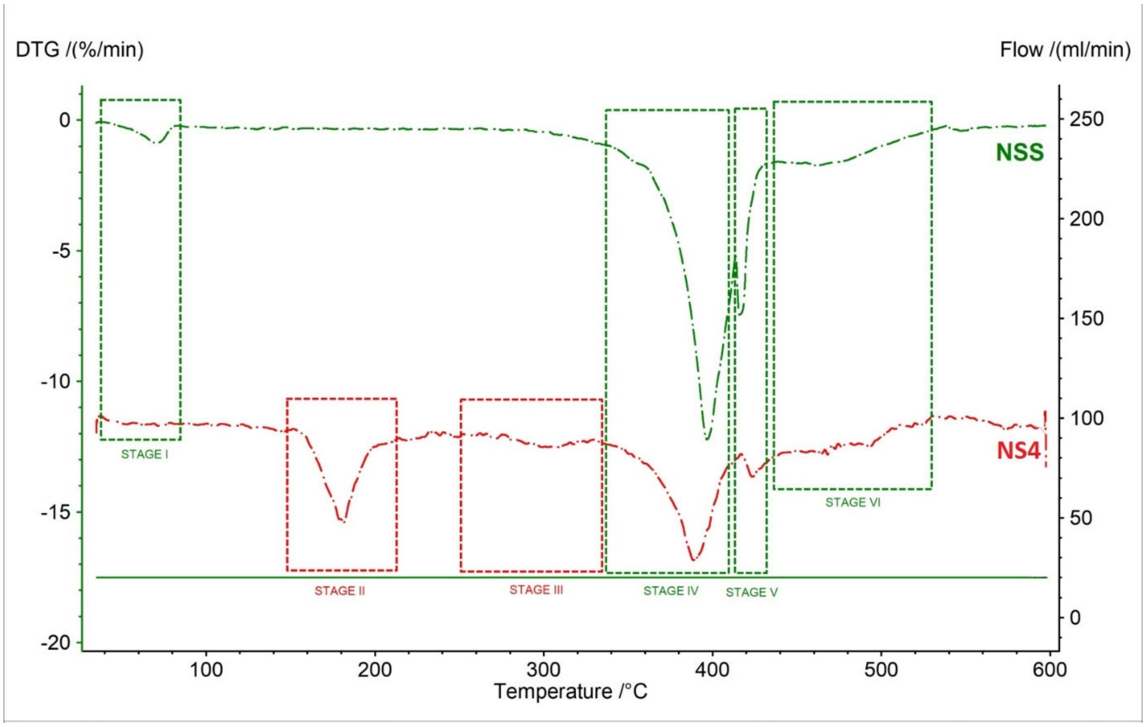

Figure S7. DTG curves of naproxen sodium standard (NSS), and pharmaceutical preparation containing naproxen sodium (NS4). The green frame shows the stages of decomposition related to the API, and the red frame - is associated with the excipient.

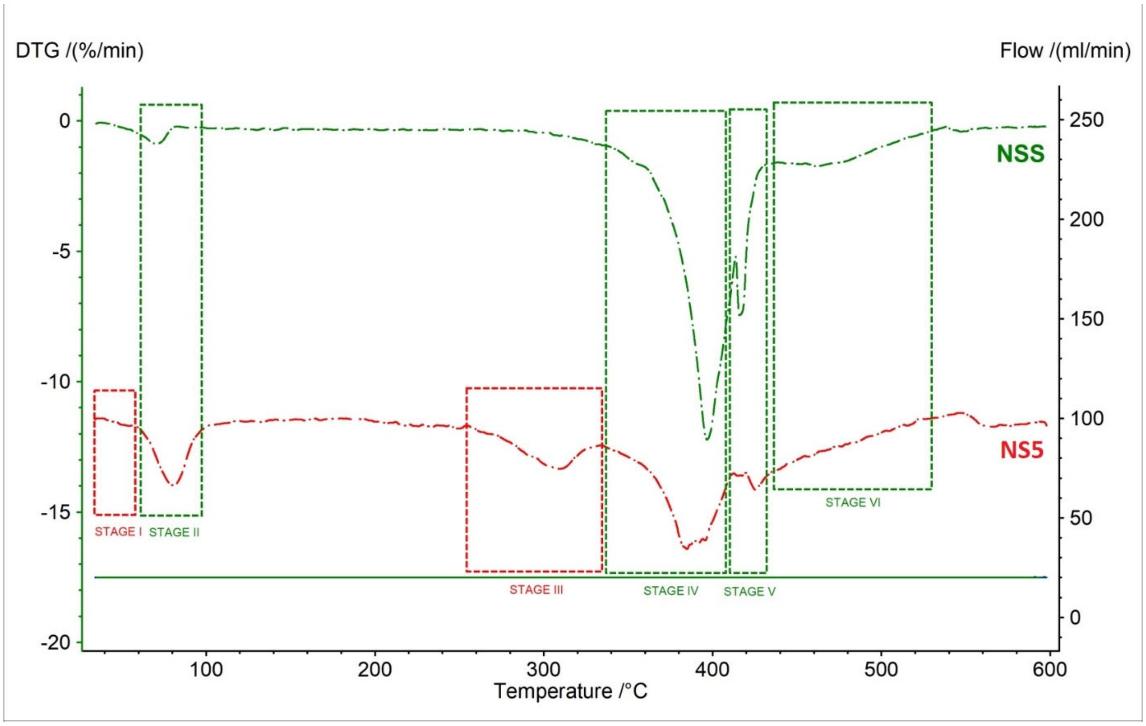

Figure S8. DTG curves of naproxen sodium standard (NSS), and pharmaceutical preparation containing naproxen sodium (NS5). The green frame shows the stages of decomposition related to the API, and the red frame - is associated with the excipient.
